# Case report: Rare restrictive cardiomyopathy with ventricular fibrillation as initial symptom rescued by automatic external defibrillator in a pediatric patient

**DOI:** 10.3389/fcvm.2022.1058341

**Published:** 2022-11-11

**Authors:** Lianfu Ji, Jinlong Chen, Yuming Qin, Shiwei Yang

**Affiliations:** Department of Cardiology, Children’s Hospital of Nanjing Medical University, Nanjing, China

**Keywords:** restrictive cardiomyopathy, ventricular fibrillation, *TNNI3*, AED, constrictive pericarditis

## Abstract

Restrictive cardiomyopathy (RCM) is a rare form of heart muscle disease with poor prognosis. Its primary manifestations were caused by systemic or pulmonary circulation congestion. Here, we reported a case of RCM with ventricular fibrillation as initial symptom in a 7-year-old boy. The child suffered cardiac and respiratory arrest suddenly while exercising at school and immediately was given external chest compression and defibrillation by the school’s equipped automatic external defibrillator (AED). The rescue was successful. At the time of the AED discharge, his electrocardiogram (ECG) indicated ventricular fibrillation. Upon further examination, the echocardiogram revealed enlarged bilateral atria, decreased diastolic function and normal ventricular thickness. Genetic analysis identified a heterozygous missense mutation [c.611(exon8)G>A,p.R204H] of *TNNI3* in the proband boy. This case contributes to our understanding of RCM in children and emphasizes the importance of having AEDs available in public places.

## Introduction

Restrictive cardiomyopathy (RCM), a rare type of cardiomyopathy, is characterized by diastolic dysfunction, enlargement of the left atrium or bilateral atria and normal or nearly normal ventricular thickness (1–3). Typically, systolic function is normal or nearly normal, but in advanced stages of RCM, it may decrease (4). The clinical manifestations of RCM which were primarily caused by systemic or pulmonary circulation congestion were non-specific and varied. Pediatric patients with RCM may suffer dyspnea, distended jugular veins, large liver, respiratory rales, ascites, lower limb edema, and even syncope (1, 3). Syncope may be a sign of low cardiac output ischemic, arrhythmias or sudden death (5).

Genetic and non-genetic factors contribute to RCM. Pathogenic mutations in over 19 different genes related to RCM have been identified (6). The major mutations are found in genes encoding for sarcomere proteins, such as cardiac troponins I (*TNNI3*), alpha tropomyosin (*TPM1*), titin (*TTN*), and so on (7). Mutations in non-sarcomeric genes are also relevant, for example in the filamin-C (*FLNC*), αB-crystallin (*CRYAB*), and desmin (*DES*) (8–11). Here we reported a case of RCM with ventricular fibrillation in a 7-year-old boy who was successfully rescued by AED outside the hospital.

## Clinical presentation

A 7-year-old boy was admitted to our emergency department after losing consciousness for an hour. The boy suffered a sudden cardiac and respiratory arrest 1 h ago while exercising at school. He was immediately given external chest compression and defibrillation used AED equipped in the school. As a result, his heartbeat and respiration recovered. When the boy was sent to our hospital, he was conscious and agitated which was alleviated by sedation and intracranial pressure reduction. His vital signs were measured: heart rate 124/min; respiratory rate 25/min; blood pressure 102/55 mmHg. On his physical examination, the liver and spleen were not touched under the ribs and there was no edema in the lower limbs. The boy and his parents were in generally good health and other family members denied a history of syncope, sudden death or cardiomyopathy.

**FIGURE 1:**
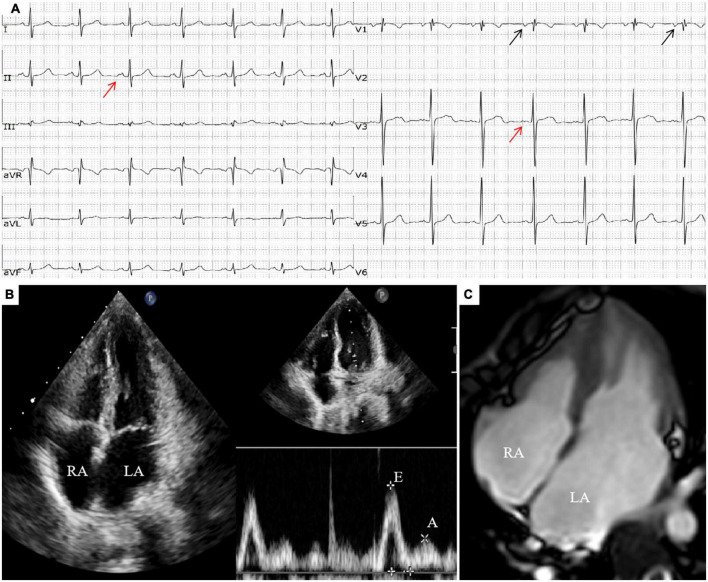
**(A)** ECG showed sinus rhythm, incomplete right bundle branch block, Ptf-V_1_ < –0.04 mm⋅s (the black arrow) and biphasic P waves (the red arrow). **(B)** Echocardiogram showed biatrial enlargement, left atrium obvious (LA: 32 mm, RA: 30 mm) and decreased diastolic function (E/A = 2.6). **(C)** Transverse section of CMR showed biatrial enlargement slightly.

**FIGURE 2:**
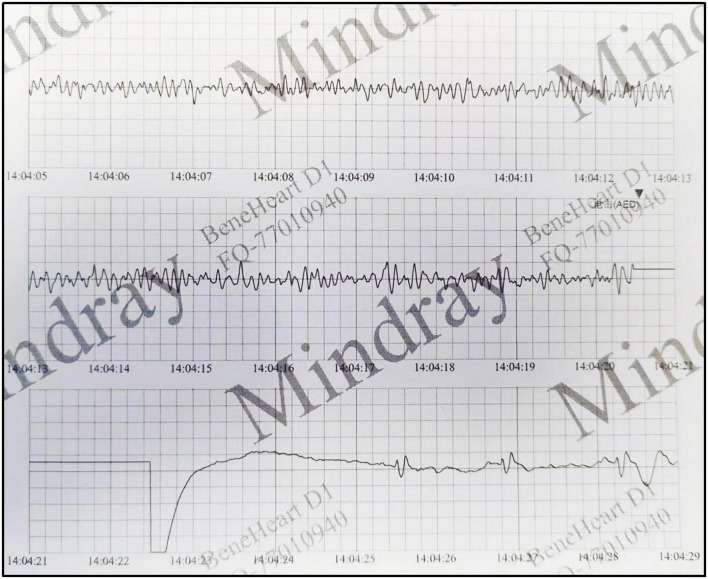
The ECG at the time of the AED discharge indicated ventricular fibrillation.

There were no obvious abnormalities in the brain MRI and video electroencephalogram (VEEG). The electrocardiogram (ECG) revealed Ptf-V_1_ < –0.04 mm⋅s and biphasic P waves, indicating left atrial enlargement and the chest X-ray revealed an enlarged heart shadow. The echocardiogram showed biatrial enlargement, especially in the left atrium (LA: 32 mm, RA: 30 mm), decreased diastolic function (E/A = 2.6; E Peak deceleration time 130 ms; Constant volume diastolic time 49 ms), moderate mitral valve regurgitation and left ventricular ejection fraction of 51.8%. Except for bilateral enlargement, cardiac magnetic resonance (CMR) indicated no abnormalities (see Figure 1). The ECG at the time of the AED discharge indicated ventricular fibrillation (see Figure 2). Troponin T and brain natriuretic peptide (BNP) levels were normal.

**FIGURE 3:**
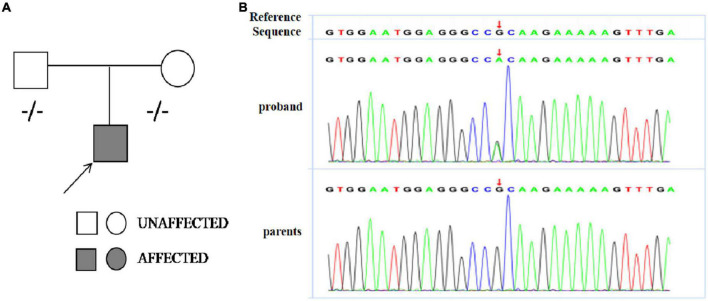
**(A)** Pedigree analysis of the family of patient. The arrow points out the proband. Circles correspond to female. Squares correspond to male. The mutation was indicated –/– if negative. **(B)** Sanger sequencing revealed a heterozygous missense mutation [c.611(exon8)G>A,p.R204H] of *TNNI3* in the proband boy and his parents did not carry the mutation.

Genetic analysis revealed the boy had a heterozygous missense mutation [c.611(exon8)G>A,p.R204H] of *TNNI3* which was not detected in his biological parents and *de novo* (see Figure 3). This mutation located in exon 19 of *TNNI3* which was conserved. We analyzed the mutation using bioinformatics protein function prediction software PolyPhen_2 and Mutation Taster and found the variant is possibly detrimental. Common genetic mutations in arrhythmias such as *SCN5A, KCNH2, KCNQ1, CACNA1C, RYR2*, and *LMNA* (12) were not found in the family members.

According to the results above, we concluded that the boy was a patient of primary RCM. At present, the vital signs of the boy are stable. We communicated with the parents and suggested installing an implantable cardioverter defibrillator (ICD) for the boy, but the parents refused. We have informed his parents that a heart transplant may be required if the disease progresses further. At present, the boy is being treated with oral diuretics and metoprolol tartrate tablets and he is in outpatient follow-up.

## Discussion

Restrictive cardiomyopathy (RCM) is the least common phenotype among pediatric heart muscle diseases. Despite accounting for only about 5% of all diagnosed cardiomyopathies, RCM has the worst prognosis. In children, half of all deaths occurred within 2 years after diagnosis and heart transplantation is frequently the only treatment (1, 3).

To confirm the diagnosis of RCM, a series of examinations should be performed in addition to the relevant clinical manifestations. Echocardiography showed left atrial or biatrial enlargement and decreased ventricular diastolic function identified by increased parameters of early diastolic velocity (E wave) and low late filling velocity (A wave), mitral inflow E/A ratio > 2.5 (13). Atrial enlargement and restrictive filling may cause mitral or tricuspid regurgitation. CMR can reflect the changes of cardiac structure and function in children with RCM, and clearly show the extent and scope of lesions. It is a specific and sensitive non-invasive examination method for the diagnosis of RCM at present (14). With the development of molecular genetics, more and more studies have found that genetic factors play an important role in cardiomyopathy (15). *TNNI3* gene is the first gene identified to be associated with RCM. The gene is a conserved sequence of cardiac troponin I gene, whose mutation can affect the function of troponin (16). As research advances, more and more genes related to RCM have been discovered, such as cardiac actin (*ACTC1*), cardiac myosin binding protein C (*MYBPC3*), β-myosin heavy chain (*MYH7*), titin (*TTN*), troponin T (*TNNT2*), filamin-C (*FLNC*), αB-crystallin (*CRYAB*), desmin (*DES*), etc. (7–11, 17–19). In this boy, we found no other variants in genes associated with RCM, except for *TNNI3.*

Restrictive cardiomyopathy must be distinguished from constrictive pericarditis (CP), for they have several similarities in clinical symptoms and hemodynamic changes. In addition, pediatric patients with sudden malignant ventricular arrhythmia, syncope attack, adams-stokes syndrome or sudden cardiac death should be alert to cardiac ion channel disease, such as long Q-T syndrome (LQTS), brugada syndrome, catecholaminergic polymorphic ventricular tachycardia(CPVT), etc. (1, 3). The boy had no history of infection, no pericardial thickening or calcification was observed on CMR and multiple QTc measurements were normal. Combined with the results of echocardiogram, ECG, CMR, and genetic analysis, the diagnosis of primary RCM was clear.

The boy had never felt discomfortable before and his first symptom was ventricular fibrillation which is rare in RCM. Biatrial enlargement and left ventricular stiffness may contribute to the arrhythmias (20). This case not only deepens our understanding of RCM in children, but also highlights the importance of AED placing in public places. Timely defibrillation with an AED was the key to saving his life when the boy suffered a sudden cardiac and respiratory arrest in school. AEDs, also known as “life-saving devices,” are portable medical devices that can be used by non-medical personnel to restore the heart rhythm of patients experiencing cardiac arrest or ventricular fibrillation and prevent sudden cardiac death. In recent years, there have been an increasing number of reports about saving lives in airports, schools, and railway stations by AED which reflects the effectiveness of strengthening the installation and training of AED in public places in China.

## Data availability statement

The raw data supporting the conclusions of this article will be made available by the authors, without undue reservation.

## Ethics statement

The studies involving human participants were reviewed and approved by the Ethics Committee of Children’s Hospital Affiliated to Nanjing Medical University. Written informed consent to participate in this study was provided by the participants’ legal guardian/next of kin. Written informed consent was obtained from the minor(s)’ legal guardian/next of kin for the publication of any potentially identifiable images or data included in this article.

## Author contributions

LJ edited the manuscript. JC contributed to samples collection. YQ and SY revised the manuscript. All authors contributed to the article and approved the submitted version.
